# Amphiregulin (AREG) and Epiregulin (EREG) Gene Expression as Predictor for Overall Survival (OS) in Oxaliplatin/Fluoropyrimidine Plus Bevacizumab Treated mCRC Patients—Analysis of the Phase III AIO KRK-0207 Trial

**DOI:** 10.3389/fonc.2018.00474

**Published:** 2018-11-08

**Authors:** Sebastian Stintzing, Boryana Ivanova, Ingrid Ricard, Andreas Jung, Thomas Kirchner, Andrea Tannapfel, Hendrik Juette, Susanna Hegewisch-Becker, Dirk Arnold, Anke Reinacher-Schick

**Affiliations:** ^1^Department of Medicine III, University Hospital, Ludwig-Maximilians-University, Munich, Germany; ^2^Institute of Pathology, University of Munich, Munich, Germany; ^3^Institute of Medical Informatics, Biometry, and Epidemiology, Ludwig-Maximilians-University, Munich, Germany; ^4^Institute of Pathology, Ruhr-University Bochum, Bochum, Germany; ^5^Hematological and Oncological Practice Eppendorf (HOPE), Hamburg, Germany; ^6^Asklepios Tumorzentrum Hamburg, Asklepios Klinik Altona, Hamburg, Germany; ^7^Department of Hematology, Oncology and Palliative Care, St Josef-Hospital, Ruhr University Bochum, Bochum, Germany

**Keywords:** colorectal cancer, maintenance, oxaliplatin, 5-FU, Bevacizumab, amphiregulin, epiregulin

## Abstract

**Background:** The EGFR (epithelial growth factor receptor) ligands amphiregulin (AREG) and epiregulin (EREG) have been considered as predictors for EGFR-antibody efficacy. The effect of AREG and EREG expression levels in primary tumor samples on the outcome of bevacizumab-treated patients is unknown.

**Patients and Methods:** Formalin-fixed paraffin-embedded (FFPE) tumor samples from surgically removed primaries of the AIO KRK-0207 trial have been tested for AREG and EREG expression. The AIO KRK-0207 trial was a randomized phase-3 study to investigate the best maintenance strategy after oxaliplatin/fluoropyrimidine plus bevacizumab induction treatment in patients with mCRC. Association of AREG and EREG levels with outcome parameters were investigated, taking into account RAS and BRAF mutations.

**Results:** A total of 331 tumor samples had measurable AREG and EREG tissue levels. In the total cohort using continuous expression levels, higher logAREG and logEREG levels were associated with a significant longer overall survival (OS) (HR 0.80; *p* = 0.003 and HR 0.78; *p* = 0.001, respectively). The subgroup of BRAF mutant tumors displayed significantly lower AREG and EREG levels compared to wild-type tumors. The prognostic effect of AREG and EREG expression was limited to the double wild-type subpopulation, whereas in the RAS mutant and BRAF mutant subgroups no prognostic effect was detected.

**Conclusion:** Low logAREG and logEREG levels are associated with a shorter OS in oxaliplatin/fluoropyrimidine plus bevacizumab treated patients. As low AREG and EREG level are associated with BRAF mutations, the prognostic value of EREG and AREG levels is limited to the RAS and BRAF wild-type subpopulation.

## Introduction

According to current guidelines, testing for RAS and BRAF (V600E) mutations (1, 2) is recommended before initiating treatment in metastatic colorectal cancer (mCRC). EGFR (epithelial growth factor receptor) antibodies are not efficient in RAS (rat sarcoma) mutant tumors (3) and may even have a detrimental effect on overall survival (OS) if used in combination with oxaliplatin (4, 5). Thus, the use of the anti-EGFR-antibodies cetuximab and panitumumab is restricted to patients with tumors not carrying a RAS mutation. Besides this negative predictive value of RAS mutations for anti-EGFR strategies both RAS and BRAF mutations have been found to be prognostic for OS (6). In fact, a V600E mutation in BRAF is one of the most detrimental prognostic factors in mCRC. This observation is limited to microsatellite stable (MSS) cases (7) whereas BRAF mutant microsatellite instable cases (MSI) have a prognosis similar to BRAF wildtype patients.

In the quest to further define predictive biomarkers to guide the treatment in mCRC, despite extensive research neither gene expression levels or gene-expression based scores nor the lately published CMS (consensus molecular subgroup) classification (8) have been introduced into clinical practice, yet. Expression of the EGFR ligands amphiregulin (AREG) and epiregulin (EREG) has been investigated with regard to their predictive value for the use of anti-EGFR antibodies (9) as even in RAS wild-type tumors only about 70% of patients respond to anti-EGFR treatment according to RECIST (response evaluation criteria in solid tumors) (10). Consequently, a number of studies showed that high AREG and/or EREG expression levels are predictive for a benefit from anti-EGFR treatment with higher response rates, longer PFS and longer OS obtained when compared to patients with low AREG/EREG expressing tumors (11–13). This finding has mainly been attributed to the molecular mechanism of competitively binding the EGFR receptor by the EGFR-antibodies cetuximab and panitumumab. Subsequently, AREG and/or EREG expression did not predict efficacy of anti-EGFR substances in RAS mutant tumors (12–14). For patients treated without anti-EGFR antibodies, a retrospective analysis demonstrated a positive prognostic value for AREG and/or EREG high expressing tumors being associated with longer OS when compared to low expressing tumors (15). The underlying mechanism has not been fully understood as higher EREG and AREG expression levels should lead to an activated EGFR pathway potentially linked to an increase in proliferation, metastasis, angiogenesis and less apoptosis (15), eventually determining shorter survival.

So far, there are no data of the prognostic or predictive value of AREG and EREG expression levels with chemotherapy alone or plus bevacizumab. As an activated EGFR pathway may induce VEGF (vascular endothelial growth factor) dependent angiogenesis (15), high AREG and EREG levels may represent a potential biomarker of bevacizumab efficacy in RAS wild-type tumors. Therefore, we retrospectively investigated samples from a phase III study using bevacizumab plus a fluoropyrimidine/oxaliplatin backbone in first-line treatment of mCRC to investigate the potential prognostic and predictive value of AREG and EREG levels.

## Patients and methods

### Study design and treatment schedule

The AIO KRK-0207 (NCT00973609) trial investigated the impact of different maintenance treatment strategies (with fluoropyrimidine (FP) plus bevacizumab, or bevacizumab alone, or no maintenance) after 24 weeks of induction treatment with a FP, oxaliplatin and bevacizumab. A total of 472 mCRC patients not progressing after 24 weeks of treatment underwent randomization into one of the treatment arms (16). The study demonstrated a non-inferiority of bevacizumab alone to FP plus bevacizumab as maintenance treatment with respect to Time to failure of strategy. For the current analysis formalin fixed paraffin embedded (FFPE) samples of the primary tumors were used to retrospectively analyze AREG and EREG mRNA levels. Inclusion criterion of the AIO KRK-0207 trial was histological proven adenocarcinoma of the colon or rectum. The reporting recommendations for tumor marker prognostic studies (REMARK) were followed (17).

### mRNA expression measurement

AREG and EREG mRNA expression was measured as published before (15). Tumor tissue was microdissected and RNA was isolated using RNeasy FFPE tissue and RNeasy MinElute CleanUp Kits (both Qiagen, Hilden, Germany) according to the manufacturers' protocol. RNA concentration was determined using a NanoDrop ND-1000 device. Reverse Transcription (RT) of RNA into cDNA was carried out using RevertAid First Strand Synthesis kit (Fermentas, ThermoScientific, Germany). RT reactions contained 1 μg total RNA, Random Hexamer Primer, 5x Reaction Buffer, RiboLock RNAse Inhibitor, 10 mM DNTP and RevertAid Reverse Transcriptase. AREG and EREG mRNA expression and expression of the housekeeping gene GAPDH was determined in duplicates by using the Universal Probe Library together with a LightCycler 480 device (Roche, Penzberg, Germany). RNAse free water was used as negative control instead of adding the template.

Analysis of tumor samples from the AIO KRK-0207 trial was approved by the ethics committee of the Ludwig-Maximilians-University Munich (#738-16).

### Statistics

For quantification, relative gene expression values were calculated using the “delta delta” ΔΔCp method, comparing pairs of AREG or EREG, respectively with the minimum value pair of the whole measurement series. Median relative expression levels of AREG and EREG were used to define high and low expression levels, respectively. Cut-offs were recalculated for each molecularly defined subgroups such as RAS mutant, RAS wild-type, BRAF mutant and BRAF and RAS wild-type. Associations of RAS and BRAF mutations with AREG and EREG expression were estimated with an ANOVA analysis after log-transformation of AREG and EREG in multivariate stratified analyses. ECOG performance status, age, gender and primary tumor location were taken into account. OS and PFS, stratified by high vs. low expressing tumors were estimated using Kaplan–Meier analysis, and significance of differences was evaluated by log-rank test and COX regression analysis. Interaction effects between mutation type and log-transformed AREG/EREG on OS and PFS were assessed by performing univariate COX regression in mutation subgroups. All *p* < 0.05 (two-sided) were regarded significant. No adjustment for multiple testing was performed. SPSS PASW 23.0 (SPSS, Chicago, IL, United States), SAS 9.2 (SAS institute, Cary, NC, United States), and R (version 3.2.2) software were used for statistical analysis.

The functional forms of the influence of log AREG, log EREG on OS, PFS were investigated with the use of fractional polynomials and smoothing splines. The usefulness of a cutoff calculation for both, AREG and EREG levels with respect to survival was examined for the given study population.

## Results

**Figure 1:**
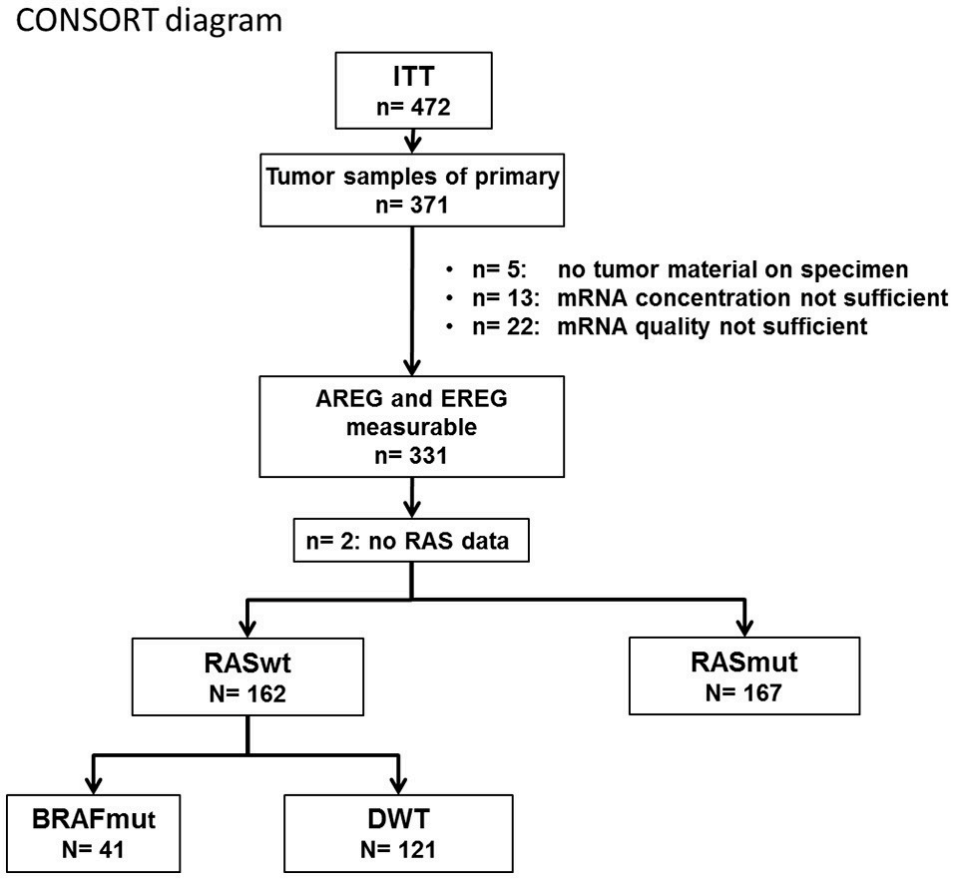
CONSORT diagram.

Out of 371 tumor samples, 331 had measurable AREG and EREG expression levels (see CONSORT diagram (Figure 1) for all subgroups). Data for both, AREG and EREG expression levels were measurable in 331 patients of the ITT. Log-transformed AREG and EREG expression were significantly correlated with each other using linear regression analysis (correlation coefficient 0.66; *p*< 2 × 10^−16^).

Using log-transformed AREG and EREG levels, we first analyzed their association with BRAF V600E and RAS mutations with the use of an ANOVA. Pairwise comparisons were adjusted for multiplicity (one step procedure). We found significantly lower AREG and EREG levels in BRAF V600E mutant samples compared to BRAF wild-type tumors and RAS mutant tumors (pairwise comparison, *p*-value adjusted for multiplicity *p* < 0.0001 for both). The association of RAS mutation and lower AREG (pairwise comparison, adjusted *p*-value *p* = 0.13) or lower EREG (pairwise comparison, adjusted *p*-value *p* = 0.011) levels when compared to RAS wild-type tumors was less obvious.

Results from the fractional polynomial procedure showed an impact of AREG and EREG on OS. The test comparing the best-fitting 2nd degree fractional polynomial against the null model led to *p*-values of 0.0004 for log AREG and 0.02 for logEREG. Moreover, it suggested that the functional form of logEREG on OS log hazard could be linear as the test comparing the best-fitting 2nd degree fractional polynomial against a straight line was not significant (*p* = 0.67). The functional form between logAREG and OS log hazard was less simple as the best model involved terms in logAREG to the power 3 multiplied by log (log AREG). However, as shown in the Figures S1, S2, the functional form looked linear decreasing for AREG values lying between about 0.01 and 0.4 defining an interval which includes 76.1% of the sample observations. The strong increase in OS log relative hazard observed for AREG values larger than 1 should be interpreted with caution. Polynomials are quite sensitive to extreme values and the influence on the model fit of the 2 patients with the largest AREG values (4.19 and 5.96) is non-negligible as both of these patients have quite short OS (20.7 and 10.3 months, respectively). This increase is less prominent in the corresponding spline fit displayed in Figure S1. Therefore, the determination a cutoff value for both AREG and EREG is not clinical meaningful.

Using log AREG and log EREG levels as continuous variable, they were also associated with primary tumor location with higher levels in left-sided primaries when compared to right-sided primaries (likelihood ratio test, *p* = 0.0003 for logAREG and *p* < 0.00001 for logEREG). This correlation was lost for logAREG when RAS and BRAF V600E wild-type tumors were analyzed (*p* = 0.25) but stayed significant for logEREG (*p* = 0.019).

We subsequently used a median split of logAREG and logEREG expression levels dichotomize data and define patients with high and low expression. Data on the distribution of baseline characteristics for these subgroups are found in Table 1. In short, looking at the whole study population there was no difference for age or ECOG. Low EREG and low AREG populations were in general more frequently right-sided and in BRAF V600E tumors (see Table 1) which is in line with the findings when AREG and EREG levels were used as continuous variables.

**Table 1:** Clinical characterization according to AREG and EREG levels.

	**All *N* = 331**	**EREG high *N* = 166**	**EREG low *N* = 165**	**AREG high *N* = 165**	**AREG low *N* = 166**	**DWT *N* = 121**	**EREG high *N* = 61**	**EREG low *N* = 60**	**AREG high *N* = 61**	**AREG low *N* = 60**	**RAS mut *N* = 166**	**EREG high *N* = 83**	**EREG low *N* = 83**	**AREG high *N* = 83**	**AREG low *N* = 83**	**BRAF mut *N* = 41**	**EREG high *N* = 21**	**EREG low *N* = 20**	**AREG high *N* = 21**	**AREG low *N* = 20**
Age (median) yrs	65	65	66	65	65	65	65	66	65	66	66	64	67	66	67	63	63	64	59	64
**ECOG (%)**																				
0	53.2	51.8	54.5	53.3	53.0	52.1	57.4	46.7	59.0	45.0	53.0	50.6	55.4	50.6	55.4	53.7	52.4	55.0	52.4	55.0
1	40.5	39.8	41.2	37.6	43.4	42.1	34.4	50.0	31.1	53.3	40.4	39.8	41.0	39.8	41.0	39.0	47.6	30.0	42.9	35.0
2	3.6	3.6	3.6	4.2	3.0	4.1	4.9	3.3	6.6	1.7	3.0	2.4	3.6	2.4	3.6	4.9		10.0	4.8	5.0
na	2.7	4.8	0.6	4.8	0.6	1.7	3.3		3.3		3.6	7.2		7.2		2.4		5.0		5.0
**COLON/RECTUM (%)**																				
Colon	67.7	57.8	77.7	59.4	75.9	59.5	57.4	61.7	52.5	66.7	68.1	60.2	75.9	60.2	75.9	90.2	81.0	100	85.7	95.0
Rectum	32.3	42.2	22.4	40.6	24.1	40.5	42.6	38.3	47.5	33.3	31.9	39.8	24.1	39.8	24.1	9.8	19.0		14.3	5.0
**SIDEDNESS (%)**																				
Right sided	32.0	20.5	43.6	26.1	38.0	16.5	13.1	20.0	13.1	20.0	34.3	25.3	43.4	31.3	37.3	70.7	66.7	75.0	66.7	75.0
Center sided	65.6	77.7	53.3	72.1	59.0	81.8	85.2	78.3	85.2	78.3	63.3	72.3	54.2	66.3	60.2	24.4	28.6	20.0	28.6	20.0
na	2.4	1.8	3.0	1.8	3.0	1.6	1.6	1.7	1.6	1.7	2.4	2.4	2.4	2.4	2.4	4.9	4.8	5.0	4.8	5.0
**MUTATIONS (%)**																				
DWT	36.6	42.8	30.3	43.6	29.5	100	100	100	100	100										
RAS mutant	50.4	52.4	67.3	52.2	48.8						100	100	100	100	100					
BRAF mutant	12.4	4.2	20.6	3.6	21.1											100	100	100	100	100
na	0.6	0.6	0.6	0.6	2.4															

*N, numbers of patients; AREG, amphiregulin; EREG, epiregulin; RAS, rat sarcoma; BRAF, RAS associated factor B; na, not available; ECOG, Eastern Cooperative Oncology Group performance status; yrs, years; mut, mutant; wt, wild-type; DWT, double wild-type (RAS and BRAF wild-type)*.

**Figure 2:**
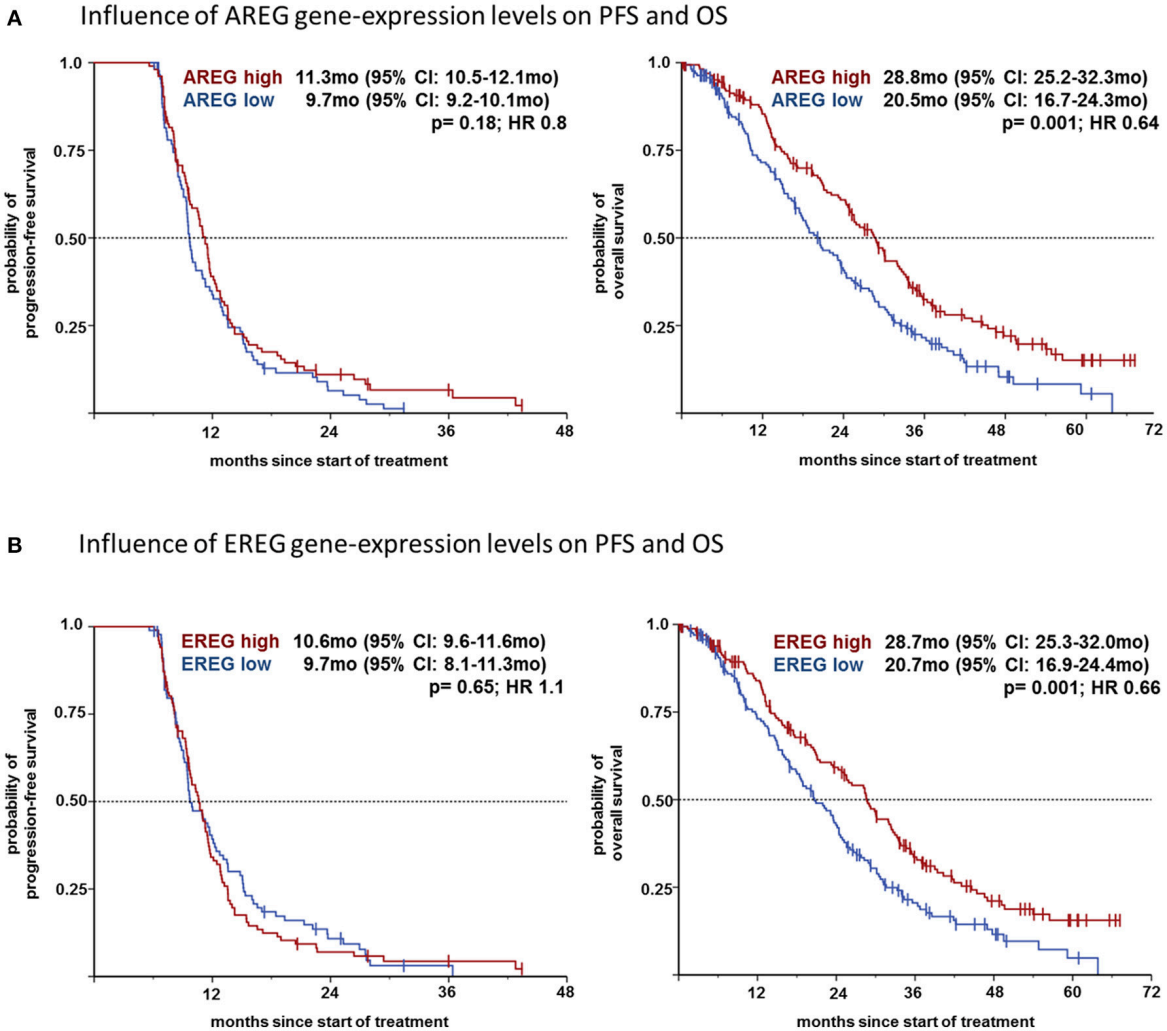
**(A)** Influence of AREG gene-expression levels on PFS and OS. **(B)** Influence of EREG gene-expression levels on PFS and OS.

We then analyzed the effect on the outcome parameters ORR, PFS and OS using dichotomized logAREG and logEREG expression levels. There was no association of AREG and EREG levels (high vs. low using the median as cutoff) with tumor response (ORR) neither in the whole study population nor in the respective mutational subgroup (see Table 2). However, high AREG and EREG levels were associated with significant longer OS in the total cohort (see Figure 2), but no difference in PFS could be detected.

**Table 2:** Overall response rate (ORR) in dependence of AREG and EREG.

		**ORR**	***p***
**AREG**			
All patients	low	47.7	0.07
	high	58.4
DWT	low	53.4	0.35
	high	63.2
RAS mut	low	50.0	0.51
	high	56.8
BRAF mut	low	27.8	0.73
	high	35.3
**EREG**			
All patients	low	50.0	0.36
	high	56.0
DWT	low	58.6	>0.99
	high	57.9
RAS mut	low	53.4	>0.99
	high	53.3
BRAF mut	low	42.1	0.17
	high	18.8

*AREG, amphiregulin; EREG, epiregulin; high, ≥median expression level; low, <median expression level; ORR, overall response rate; p, two-sided Fisher's exact test p; DWT, double wild-type (RAS and BRAF wild-type*.

**Figure 3:**
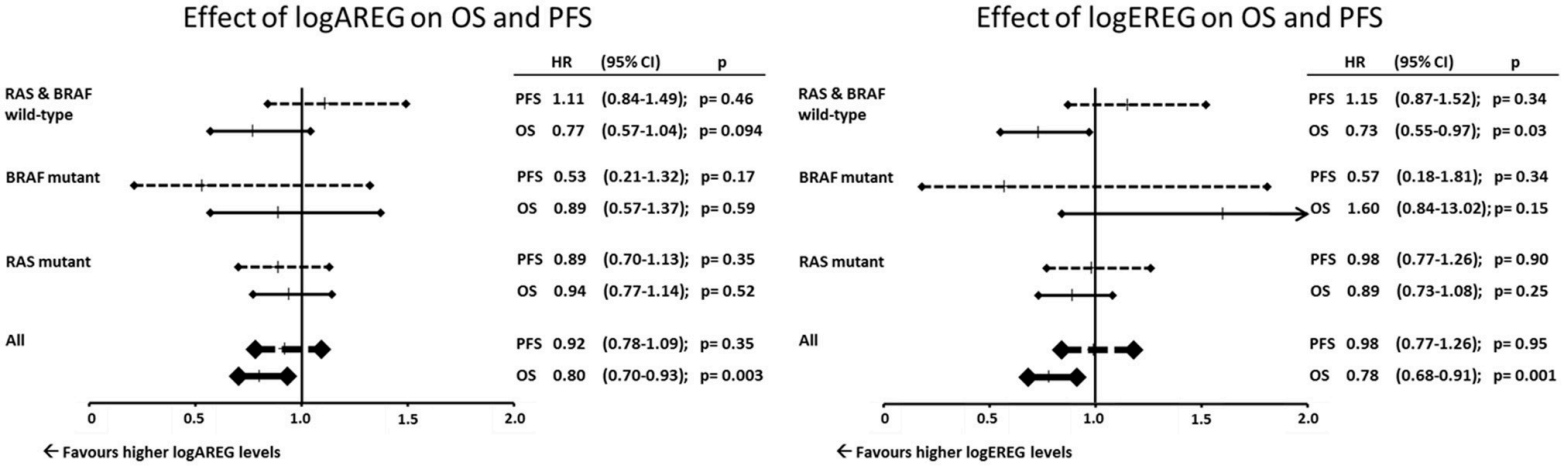
Effect of logAREG on OS and PFS. Effect of logEREG on OS and PFS.

Due to the association of BRAF V600E mutation with AREG and EREG a Cox regression analysis using EREG and AREG levels as log-transformed continuous variables was performed and adjusted to age, gender and primary tumor location. Here the prognostic effect on OS for both, AREG and EREG was limited to the BRAF and RAS wild-type subpopulation (Figure 3). Additionally AREG and EREG levels were analyses according to MSI. In total only 28 patients had a MSI tumor and no association with AREG and EREG levels were found (data not shown)

**Figure 4:**
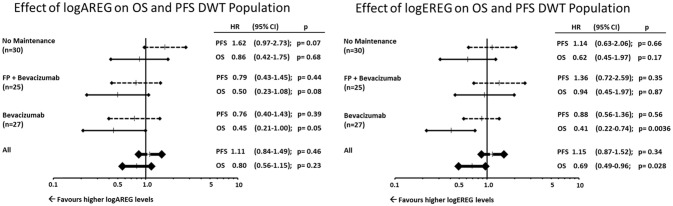
Effect of logAREG on OS and PFS DWT population. Effect of logEREG on OS and PFS DWT population.

Furthermore, the associations or AREG and EREG levels were analyzed with regard to the respective maintenance arms. Within the double-wildtype population (RAS and BRAF wildtype) only the bevacizumab maintenance arm showed a significant association with EREG and AREG levels with regard to OS (Figure 4).

## Discussion

The current analysis demonstrates for the first time a prognostic value of high AREG and EREG expression levels in patients undergoing a first-line combination treatment including oxaliplatin, fluoropyrimidine and bevacizumab. Due to the almost linear distribution of AREG and EREG levels with regard to survival, it is not feasible to calculate a robust and consistent cut-off. Both, EREG and AREG expression levels were significantly lower in BRAF mutant tumors but not in RAS mutant tumors. Also, in double wild-type tumors, left sided primaries had higher EREG levels than right-sided tumors which is in line with the finding that left-sided tumors have a better outcome when compared to right-sided tumors. After adjustment for known prognostic factors, this association of expression with better OS was limited to the double-wildtype (RAS and BRAF wild-type) subpopulation. As no effect on response rates and PFS could be established, the differences of efficacy according to AREG and EREG levels may also be attributed to further-line treatment.

AREG and EREG expression levels were associated negatively with BRAF mutations. This has been reported previously (11). As both, RAS and BRAF mutations lead to a constitutively active EGFR pathway which is independent of exocrine signaling through the EGFR ligands AREG and EREG (18) the underlying mechanism and distinct roles of RAS and BRAF in mCRC still have to be elucidated in more detail. RAS and BRAF mutations differentially regulate cellular hierarchies, stem cell function and mCRC development (19). RAS mutant mCRC are mostly chromosomal instable (CIN), MSS (micro-satellite stable) tumors associated with mutant APC and do not respond to anti-EGFR treatment (2). BRAF mutant mCRCs on the other hand are associated with MSI (microsatellite instability), CIMP (CpG-island methylation phenotype detection), and the expression of -catenin to the nucleus (19). We were not able to find an association of AREG or EREG levels with respect to MSI. Although within the BRAF V600E mutant subgroup of tumors, no associations with respect to AREG/EREG levels could be established. As the numbers were rather small, this may be due to limited power of our analysis. It has been shown that BRAF V600E mutations in contrast to KRAS mutations lead to a silencing of genes by MAFG (transcription factor MafG), BACH (transcription regulator gene BACH) and epigenetic modifiers such as CHD8 (chromodomain-helicase-DNA-binding protein 8) and DNMT3B (DNA (cytosine-5-)-methyltransferase 3 beta) (20). This may, at least in part, explain the significantly lower AREG and EREG levels of BRAF mutant tumors in our analysis.

EREG and AREG levels have been shown to predict tumor response and disease control in patients treated with EGFR-antibodies (11, 13, 14, 21) in both, RAS or KRAS exon 2 wild-type populations. This correlation could not be demonstrated in the current analysis of oxaliplatin/fluoropyrimidine bevacizumab treated patients (see Table 2). As neither oxaliplatin nor fluoropyrimidine nor bevacizumab is targeting the EGFR dependent pathway (22) this result could have been anticipated, but somehow is in conflict with previous published observations showing an association with PFS and OS in chemotherapy only treated patients (15). Within the chemo-only trial (15) all patients had been treated with irinotecan and therefore it would be of interest whether the effect on PFS is associated with the chemotherapeutic backbone as AREG and EREG levels within the AIO-KRK-0207-study have no effect on PFS. This again differs from studies analyzing AREG and EREG expression in trials using EGFR antibodies (11, 13, 21) or chemotherapy only (15). As randomization took place 6 months after initiation of treatment and PFS was defined as time from randomization, data was only available for the period >6 months, which may have influenced the results of AREG and EREG on PFS.

The exploratory analyses within the double-wild-type population suggest longer PFS in bevacizumab maintenance is associated with higher AREG levels whereas in the watch- and wait-arm higher AREG levels showed a trend toward shorter PFS (Figure 4). Due to the rather small sample sizes this finding warrants further, independent validation in larger cohorts.

In our study OS was markedly different by AREG and EREG expression levels. However, it remains unclear whether the OS data have to be attributed to second- and further-line effects or to the trial design (16). AREG and EREG levels are able to define groups of patients with significantly different OS in patients with RAS and BRAF wild-type tumors under a fluoropyrimidine/oxaliplatin/bevacizumab regimen. This is in accordance with all previous published data defining both-AREG and EREG gene expression levels -, as prognostic (11, 13, 15, 21). Furthermore, patients with RAS WT may have undergone subsequent treatment with anti-EGFR compounds which may interact with the observations for OS.

As the prognostic effect of AREG and EREG levels are correlated to BRAF and RAS mutations, the question arises whether low AREG or low EREG expression levels can be seen as surrogate biomarkers for an auto-activated (e.g., by mutations) EGFR pathway. We did not test for PIK3CA mutations, neither do we have data on PTEN or cMET expression. But those have also been reported among others to be signs of an activated EGFR pathway independent of EGFR –ligand binding (23, 24).

The current analyses are limited by their retrospective nature and the number of available specimen. Furthermore, all analyzed patients also have been randomized to one of the maintenance arms. Therefore, PFS events for patients progressing early under oxaliplatin/fluoropyrimidine/bevacizumab are missing and all PFS data have to be interpreted with caution.

## Conclusion

The current analyses demonstrate the prognostic value of AREG and EREG expression levels for fluoropyrimidine/oxaliplatin/bevacizumab treated patients in first-line mCRC. This prognostic value is limited to the BRAF and RAS wild-type population. As BRAF mutations are significantly and negatively associated with AREG and EREG levels, low AREG or EREG levels may be a sign for an EGFR pathway not altered by any activating mutations and therefore have a better outcome. This is supported by the observation that within the group of patients bearing a RAS mutation neither AREG nor EREG levels were found to be of prognostic value. AREG and EREG data should therefore always be interpreted in the context of BRAF and RAS mutational status.

## Author contributions

SH-B, DA, and AR-S conduction of the clinical study. SS, BI, AJ, TK, AT, SH-B, DA, and AR-S writing. IR statistics. SS, BI, AJ, AT, and HJ translational workup.

### Conflict of interest statement

HJ received fees from BMS, MSD and Roche for giving talks and a fee from BMS for taking part in an advisory board meeting. SS received honoraria for talks and advisory boards from: Amgen, Bayer, Lilly, Merck KGaA, Sanofi, Roche, Takeda. Funding for the translational part came from Roche Pharma AG, Germany (ML21972), Grenzach-Whylen Germany. The remaining authors declare that the research was conducted in the absence of any commercial or financial relationships that could be construed as a potential conflict of interest.
