# Supplementary figures and images for: Amphiregulin (AREG) and Epiregulin (EREG) Gene Expression as Predictor for Overall Survival (OS) in Oxaliplatin/Fluoropyrimidine Plus Bevacizumab Treated mCRC Patients—Analysis of the Phase III AIO KRK-0207 Trial
Source: Front Oncol. 2018 Nov 8;8:474. doi: 10.3389/fonc.2018.00474 (PMC6236022; doi:10.3389/fonc.2018.00474)

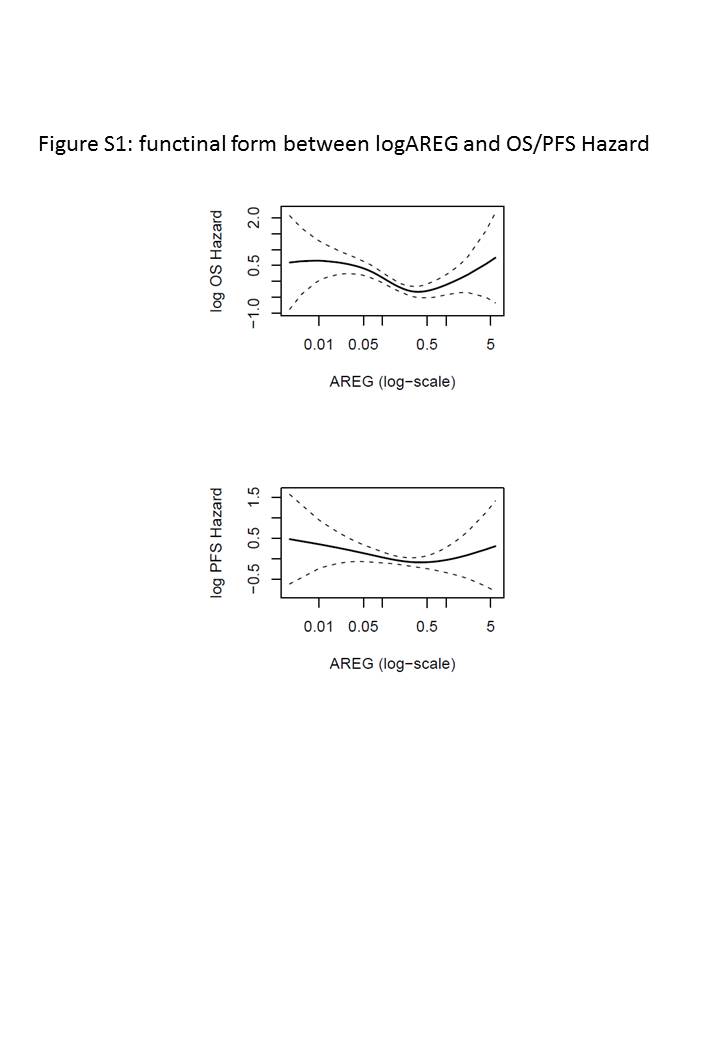

Supplement: Supplementary file 1 [file Image_1.jpg]

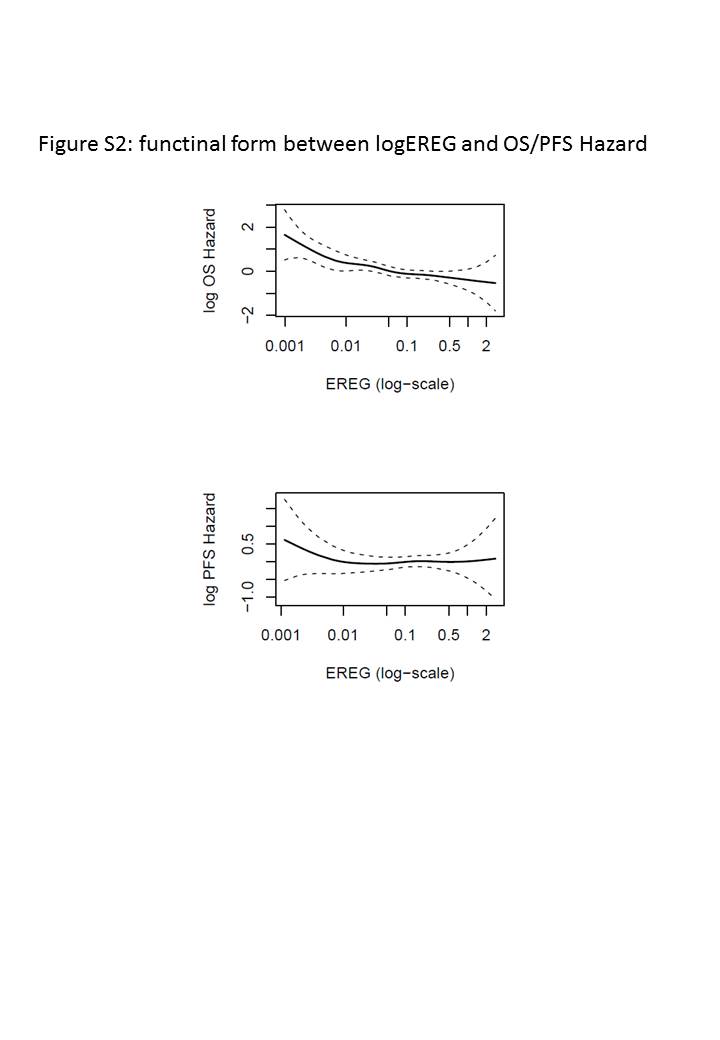

Supplement: Supplementary file 2 [file Image_2.jpg]
